# Precision Editing of NLRS Improves Effector Recognition for Enhanced Disease Resistance

**DOI:** 10.1002/advs.202511685

**Published:** 2026-01-18

**Authors:** Vinit Kumar, Vishwa I. P. W. A. Kumara, Pradeepa C. G. Bandaranayake, Dong Yawen, Yang‐Yang Gao, Junfeng Liu, Ge‐Fei Hao, Xiu‐Fang Xin

**Affiliations:** ^1^ State Key Laboratory of Green Pesticide Key Laboratory of Green Pesticide and Agricultural Bioengineering Center For Research and Development of Fine Chemicals, Ministry of Education Guizhou University Guiyang P. R. China; ^2^ Department of Physiotherapy Vinoba Bhave University Hazaribagh Jharkhand India; ^3^ Agriculture Biotechnology Centre Faculty of Agriculture University of Peradeniya Colombo Sri Lanka; ^4^ School of Pharmaceutical Sciences Guizhou University Guiyang P. R. China; ^5^ State Key Laboratory of Maize Bio‐Breeding Joint International Research Laboratory of Crop Molecular Breeding, Ministry of Agriculture Key Laboratory for Crop Pest Monitoring and Green Control China Agricultural University Beijing P. R. China; ^6^ State Key Laboratory of Green Pesticide Central China Normal University Wuhan P. R. China; ^7^ National Key Laboratory of Plant Molecular Genetics CAS Center for Excellence in Molecular Plant Sciences Institute of Plant Physiology and Ecology Chinese Academy of Sciences Shanghai P. R. China

**Keywords:** crop protection, disease resistance, effector recognition, NLR bioengineering, plant immunity, resistosome engineering

## Abstract

Plant pathogens pose a significant threat to global food security by causing up to 80% agricultural yield losses. Nucleotide‐binding, leucine‐rich repeat immune receptors (NLRs) were widely proved to protect plants from a wide array of pathogens evasion. Recent studies have shown significant progress in bioengineering NLRs to enhance plant immunity through improved pathogen recognition, investigation of immune evasion, and structural insights into NLR functions. However, bioengineering NLRs for enhanced plant immunity faces key challenges of maintaining specificity, addressing pathogen evolution, and minimizing autoactivity risks. Here, we synthesize recent advances in understanding NLR biology and highlight key bioengineering strategies, including mismatched pairs, domain swapping, and targeted mutagenesis, that leverage this knowledge to enhance disease resistance. The successful applications of NLRs precision editing strategies have also been demonstrated to improve resistance in various plants, showcasing their overall effectiveness. This review highlights promising avenues to strengthen plant immunity against pathogens, which have enormous potential for application in agriculture and breeding techniques.

## Graphical abstract

Precision engineering of plant NLR immune receptors enables rational design of enhanced pathogen resistance through mismatched pairing, domain swapping, and targeted mutagenesis. These approaches achieve multi‐fold expansion in recognition breadth while minimizing autoimmunity risks and fitness penalties. Integration of structural biology with Artificial Intelligence (AI) design tools provides actionable pathways for developing durable, broad‐spectrum crop protection addressing global food security challenges.

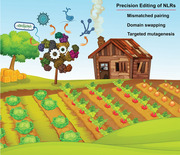

## 1 Introduction

Plant pathogens cause recurrent epidemics, endangering agricultural production and global food security [1]. Among these, pathogens such as fungi, oomycete and plant‐parasitic root‐knot nematodes (RKNs) cause devastating crop losses, with fungi reducing yields by 20–25% and RKNs causing 50–70% marketable losses valued at over 80 billion US dollars annually [2, 3, 4]. These pathogens weaken plant immunity, causing up to 40% yield loss in major crops. To counter this, plants have evolved intracellular nucleotide‐binding, leucine‐rich repeat receptors (NLRs) that recognize pathogen effectors [5, 6]. Upon recognition, NLRs trigger effector‐triggered immunity (ETI), leading to a hypersensitive response (HR) that can contain the pathogen and prevent its spread within the plant [7]. Understanding these NLR‐mediated defense mechanisms is essential for developing effective strategies to protect crops and ensure global food security.

Recently, significant advances in understanding NLR structure, regulation, and activation mechanisms have revolutionized engineering approaches [8, 9]. Earlier attempts to engineer NLRs relied on random mutagenesis and unguided domain swapping to expand pathogen recognition. These methods achieved limited success but faced major problems. Random mutagenesis required screening thousands of variants with unpredictable results and often produced autoimmune NLRs that reduced crop yields. Domain swapping without structural knowledge was inefficient, with most modified receptors either non‐functional or inappropriately activated [10]. Without understanding NLR molecular mechanisms, rational design remained impossible [11]. Recent structural biology breakthroughs, including cryo‐EM resistosome structures, effector–NLR interaction data, and regulatory insights, now enable precision engineering strategies that overcome these limitations.

Precision NLR engineering addresses these limitations through rational, structure‐guided molecular design. Precision engineered approaches enable targeted domain modifications, mismatched receptor pairing, and interface redesign that expand pathogen recognition breadth 3‐5‐fold while maintaining regulatory control [6, 12]. For example, engineered rice Pik‐1/Pik‐2 mismatched pairs recognize multiple AVR‐Pik effector variants (AVR‐PikD, AVR‐PikE, AVR‐PikF) through strategic interface modifications [13]. Similarly, potato Immunity 2 ‐ Isoleucine‐to‐Asparagine at position 141 (I2‐I141N) modifications expand recognition to include AVR3a variants while maintaining >90% resistance efficacy across three field seasons [14, 15]. Tomato NRC helper NLR variants engineered to evade pathogen suppression restore immunity against *Ralstonia solanacearum* effectors that disable wild‐type receptors [16]. These structural and biochemical insights reveal conserved NLR activation mechanisms involving ATP/ADP switches, resistosome oligomerization, and signaling molecule production, providing design principles applicable across diverse crop‐pathogen systems. However, critical technical bottlenecks remain: autoimmunity risks causing 5–10% yield penalties in some lines, fitness costs from enhanced immunity, pathogen suppression of engineered receptors through evolved effectors, and resistance breakdown against rapidly adapting pathogen populations. Overcoming these hurdles requires precision engineering strategies including domain modifications, allele pairing, and targeted mutagenesis that fine‐tune recognition and activation while minimizing trade‐offs to ensure resilient and durable immunity.

Here we synthesize recent advances in NLR precision engineering for enhanced disease resistance. We first establish the structural and mechanistic foundations of NLR function, including domain architecture, effector recognition mechanisms, ATP/ADP‐driven activation, and post‐translational regulation. We then examine three complementary engineering strategies: mismatched NLR pairs that exploit allelic variation to expand effector recognition, domain swapping approaches that overcome pathogen suppression, and targeted mutagenesis that fine‐tunes binding specificity. Through detailed case studies in rice, potato, and tomato, we evaluate engineering outcomes including recognition breadth, fitness costs, and field durability. Finally, we address implementation challenges including fitness‐immunity trade‐offs, pathogen evolutionary pressure, AI‐enabled design tools, and agricultural deployment strategies. By critically analyzing successes and limitations across these approaches, we identify key principles for developing durable, broad‐spectrum disease resistance that can be integrated into breeding programs for global food security.

## 2 Insights Into Plant Pathogen Defense: The Multifaceted Role of NLRs

### 2.1 Two Layers of Plant Defense and NLR Structural Organization

**FIGURE 1:**
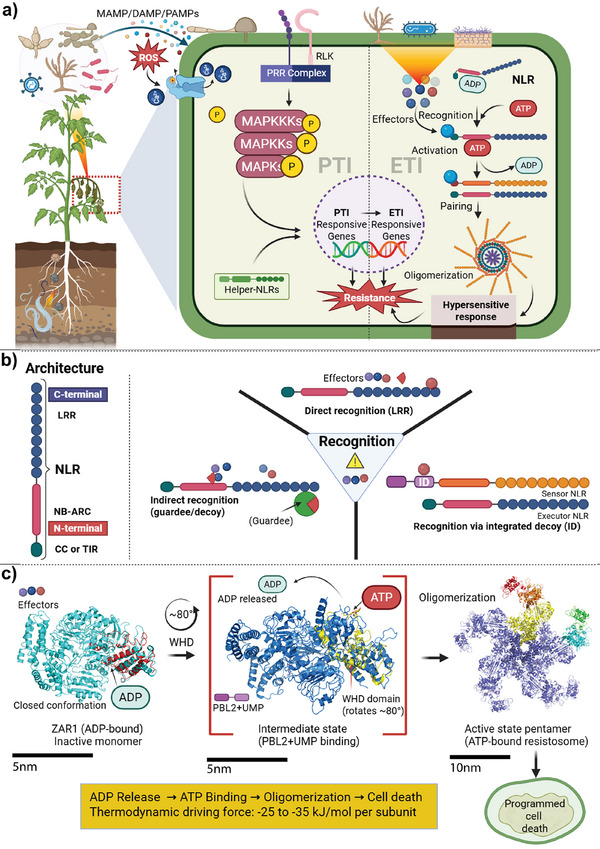
Overview of plant defense mechanisms and NLRs structure and function. (a) Two layers of plant defense against pathogens, PAMP‐triggered immunity (PTI) and Effector‐triggered immunity (ETI). PTI is activated by pattern recognition receptors (PRR) on the cell surface that detect pathogen‐associated molecular patterns (PAMPs). ETI involves intracellular resistance proteins, particularly NLRs, which recognize and counteract pathogen effectors, often causing localized programmed cell death (PCD) and hypersensitive response (HR). Together, these two layers of immunity protect plants from pathogens. (b) NLRs have a fundamental structure with Coiled coil (CC), Nucleotide‐binding (NB), and Leucine‐rich repeat (LRR) domains. They recognize effectors directly through LRR domains or indirectly via guardees or decoys. Upon pathogen recognition, inactive NLRs bound to Adenosine Diphosphate (ADP) become active by exchanging ADP for Adenosine Triphosphate (ATP), leading to oligomerization and immune responses. This complex molecular process of NLR‐mediated immune activation and pathogen identification is an essential part of plant innate immunity that aids in defense against a variety of diseases. (c) Molecular mechanism of NLR activation through ATP‐dependent conformational changes. Left: Inactive ZAR1 NLR in closed conformation stabilized by ADP binding. Middle: Effector recognition (e.g., PBL2 uridylylation by AvrAC) triggers ADP release and approximately 80° rotation of the winged‐helix domain (WHD), creating an intermediate state that facilitates ATP binding. Right: ATP binding drives oligomerization into pentameric resistosome assemblies, leading to programmed cell death and immune activation. The conformational transition from ADP‐bound monomer to ATP‐bound resistosome involves thermodynamic driving forces of −25 to −35 kJ/mol per subunit, enabling effective immune signaling while preventing autoactivation through precise nucleotide‐dependent control.

Plants employ a sophisticated defense system consisting of two interconnected layers, PTI and ETI, that work together to protect against pathogens [17]. PTI is initiated by pattern recognition receptors (PRR) on the cell surface that detect conserved pathogen‐associated molecular patterns (PAMP), activating basal defense responses [18]. ETI, in contrast, relies on intracellular NLR proteins encoded by resistance (R) genes, which recognize pathogen effectors to trigger robust immune responses (Figure 1a). NLRs belong to the STAND AAA+ ATPase superfamily, characterized by three major structural components: a central nucleotide‐binding (NB) domain, a C‐terminal leucine‐rich repeat (LRR) domain, and one or more N‐terminal domains (Figure 1b) [19, 20]. Additionally, certain NLRs contain noncanonical integrated domains (IDs) positioned at N‐terminal, C‐terminal, or internal locations, which contribute to effector recognition and broaden functional scope [21, 22]. The N‐terminal domains act as primary signaling modules and comprise two major types: the Toll/interleukin‐1 receptor (TIR) domain forming TIR‐NLRs (TNLs) and the coiled‐coil (CC) domain forming CC‐NLRs (CNLs) (Figure 1b) [23, 24]. A third class called helper or resistosome NLRs (RNLs) function downstream of sensor NLRs to execute immune signaling. N‐terminal domains mediate downstream signaling through oligomerization. The C‐terminal LRR domain is involved in effector recognition and autoinhibition, enabling recognition of diverse pathogen effectors through direct and indirect interactions [5, 25, 26]. This structural organization determines both the specificity of pathogen recognition and the subsequent signaling mechanisms employed during ETI activation.

Recent structural and biochemical studies have uncovered the molecular basis of NLR‐mediated immunity. Upon activation, TIR domains in TNLs and helper NLRs exhibit NADase enzymatic activity, catalyzing the production of signaling molecules including 2' cyclic ADP‐ribose (2′‐cADPR), pRib‐AMP/ADP, ADP‐ribose‐ATP (ADPr‐ATP), and di‐ADPR. These molecules activate downstream immune responses through complexes such as EPA (Enhanced Disease Susceptibility 1‐Phytoalexin Deficient 4–Activated Disease Resistance 1; EDS1‐PAD4‐ADR1) and EDS1‐SAG101‐NRG1 complexes, showing how TIR‐domain NADase activity generates signaling molecules that activate helper NLR pathways [27, 28]. NLR activation engages components like RIN4, effectors (AvrRpm1 and AvrB), and helper NLR (RNL) [18, 29, 30]. This ETI activation amplifies PTI responses, resulting in faster, stronger, and longer‐lasting immune reactions (Figure 1a) [31, 32]. There is a dynamic interplay between PTI and ETI, with substantial cross‐talk and overlap in their signaling pathways, highlighting the need to elucidate how PTI primes ETI or vice versa [18]. Notably, helper NLRs of the Activated Disease Resistance 1 (ADR1) family are required for basal resistance and PTI, functioning in defense regulation rather than effector detection. The EDS1‐PAD4‐ADR1 complex mediates Arabidopsis PTI via coordinated receptor signaling. Helper NLR mutants exhibit reduced resistance, demonstrating that ETI activation enhances PTI responses [33]. This dual functionality of helper NLRs positions them as critical molecular bridges connecting PTI and ETI pathways. Helper NLRs are indeed required for both immune layers: in PTI, ADR1 family members function in basal resistance independent of sensor NLR activation, while in ETI, the same helper NLRs (ADR1, NRG1) serve as downstream executors activated by sensor TNL‐produced signaling molecules (2′‐cADPR, pRib‐AMP/ADP). This convergence enables helper NLRs to integrate signals from both surface‐localized PRR (PTI) and intracellular sensor NLRs (ETI), creating a unified immune response that amplifies and sustains pathogen defense [31, 33, 34]. The ETI‐Mediating and PTI‐Inhibited Sector (EMPIS) mechanism further illustrates how PTI modulates ETI responses, shaping immune efficacy [35]. This modular architecture enables precision engineering at multiple levels. LRR domain alterations expand effector recognition (Section 2.2), NB domain modifications control activation kinetics (Section 2.3), and ID swaps introduce novel specificities (Sections 3.1 and 4.1). By targeting individual domains, engineers can independently optimize recognition, activation, and signalling without disrupting overall protein function (Table 1).

**TABLE 1:** Representative NLR immune receptors with their recognition mechanisms, effector targets, and bioengineering strategies.

NLRs	Host plant	Common effector target	Recognition mechanism	Bioengineering approach	References
ADR1‐L1, ADR1‐L2	*Arabidopsis thaliana*	AvrPtoB	Indirect: Senses EDS1 modification	Target EDS1‐PAD4‐SAG101 complex/NRG1 to reduce inhibition	[60]
Bs3, Bs4	*Solanum lycopersicum* (tomato)	AvrBs3, AvrBs4, AvrHah1	Bs3: TAL effector‐triggered executor gene activation Bs4: EDS1‐dependent TIR‐NB‐LRR signaling	Apply TALE nuclease/split‐TALE systems for genome editing	[61, 62]
Gpa2	*Solanum tuberosum* (potato)	RanBPM‐like effector (RBP1)	C‐terminal LRR domain determines effector recognition	Modify ARC2 region and N‐terminal LRR for activation control	[63]
I2, I3	*Solanum lycopersicum* (tomato)	AVR1	Direct: Binds to AVR1	Mutate/swap LRR domains to alter effector recognition	[64, 65]
L6	Flax	AvrL567	Direct: Interacts with AvrL567 effector variants	Engineer LRR domain via targeted mutations for specificity	[10, 66]
Mi‐1.1, Mi‐1.2	*Solanum lycopersicum* (tomato)	AvrBs3	Direct/Indirect: Binds to/senses GPA2 modification	Mutation of D to V in the MHD motif of the NB‐LRR	[67]
mla8	*Barley*	AvrSr50, AVRA	Direct: Interacts with pathogenic effectors	Use transgenic complementation for pathogen recognition	[68]
NbNRC2, NbNRC3	*Nicotiana benthamiana*	SPRYSEC15, AVRcap1b	Direct: Binds to SPRYSEC15	Modify domains to control oligomerization states	[69]
Prf	*Solanum lycopersicum* (tomato)	HopZ3, Lso‐HPE1	Indirect: Senses RIN4 modification	Engineer helper NLRs based on natural variation	[70, 71]
Pik‐1, Pik‐2	*Oryza sativa (rice)*	AVR‐Pik (allelic variants: AVR‐PikD, AVR‐PikE)	Direct: Pik‐1 binds AVR‐Pik via integrated Heavy Metal‐Associated (HMA domain)	Engineer HMA domain to expand recognition of MAX effectors	[72]
R3a	*Solanum tuberosum* (potato)	RipAC	Indirect: Binds host proteins	Implement single‐residue mutations for expanded response	[73, 74]
RGA5, RGA4	*Oryza sativa (rice)*	AVR‐Pia, AVR1‐CO39	Direct: RGA5 binds effectors via integrated RATX1/HMA domain	Modify RATX1/HMA domain to broaden effector recognition	[75, 76]
Roq1	*N. benthamiana*	XopQ, HopQ1	Direct: Forms tetrameric resistosome with XopQ/HopQ1	Target LRR‐effector contact residues for binding enhancement	[50]
Rpi‐blb1	*Solanum bulbocastanum* (potato)	IPI‐O4, PITG_15278, SPRYSEC10, SPRYSEC34	Direct: Binds effectors	Use cis‐genesis for R gene stacking	[77, 78, 79]
RPM1	*Arabidopsis thaliana*, *Solanum lycopersicum* (tomato)	AvrPphB, AvrRpt2	Indirect: Senses RIN4 cleavage	Modify domains for enhanced recognition/activation	[80]
RPP13	*Arabidopsis thaliana*	AvrRpt2	Indirect: Senses ATR1 modification	Study effector molecular mechanisms and targets	[81]
RPS2	*Arabidopsis thaliana*	HopF2	Indirect: Senses RIN4 modification	Optimize RPS2‐RIN4 interaction	[82]
RPS4, RPS5	*Arabidopsis thaliana*	AvrPph3, AvrRps4	Indirect: Senses PBS1 and EDS1 modification	Modify WRKY domain and phosphorylation status	[83, 84]
RPS6	*Arabidopsis thaliana*	AvrRpm1	Indirect: Senses RIN4 modification	Introduce mutations/swaps for sensitivity control	[80, 85]
Rx	*Solanum tuberosum* (potato), *Solanum lycopersicum* (tomato)	RHA1B, GrEXPB2	Direct: Binds N‐terminal domain of effector	Transform with Rpi‐amr4 gene	[86]
Sw5b	*Solanum lycopersicum* (tomato)	NSm (TSWV), SPRYSEC19, Viral Nsm	Two‐step: Domain interaction followed by NSm‐triggered activation. And SPRYSEC19 physically associates with the LRR domain	Introduce NSmRB‐responsive mutations in LRR/SD domains and Modify NB domain for hypersensitive response (HR) control and LRR for recognition	[87, 88]

### 2.2 Effector Recognition Mechanisms of Plant NLRs

Plant NLRs employ three recognition mechanisms, direct, indirect, and ID, each presenting distinct opportunities for precision engineering (Figure 1b) [36]. Direct recognition occurs when an NLR's LRR domain physically interacts with pathogen effectors. The flax L locus NLRs (L5, L6, L7) bind *Melampsora lini* AvrL567 effectors through LRR domain polymorphisms that determine recognition specificity [37], while wheat Sr50 recognizes stem rust (*Puccinia graminis* f. sp. *tritici*) AvrSr50 through similar LRR‐mediated interactions [38]. Additionally, barley Mla protects against *Blumeria graminis* f. sp. *hordei* AVRa1 via its LRR domain [39]. This interaction leads to the activation of NLRs and subsequent immune responses (Figure 1b). These examples demonstrate how LRR domain variations naturally expand recognition breadth, a principle exploited in engineering NLRs with altered effector binding specificity (Sections 3.1 and 3.3).

Indirect recognition involves NLRs monitoring host proteins (“guardees”) for effector‐induced modifications. RPS2 and RPM1 guard RIN4, detecting changes when AvrRpt2 modifies this protein to activate immunity. Similarly, RPS5 monitors the decoy protein PBS1, where AvrPphB cleavage (despite targeting the PBS1‐like kinase PBL family) triggers RPS5‐mediated immunity (Figure 1b) [40]. This is considered an expanded form of indirect recognition. This guardee‐based surveillance inspired engineering strategies that exploit decoy proteins to detect diverse effector activities (Section 3.2).

Finally, in an unconventional recognition system, ID recognition combines direct binding with molecular mimicry, where NLRs incorporate host target domains as integrated sensors [41, 42]. Guo et al., (2018) demonstrated that integrated heavy metal‐associated (HMA) domains recognize diverse MAX effectors via distinct binding surfaces, supporting the hypothesis that pathogen targets have been incorporated into NLRs [43]. For example, in rice where numerous NLRs employ HMA domains as IDs to bait pathogens and trigger immunity [44]. These NLRs typically function as sensor‐helper pairs: Pik‐1/Pik‐2 (where Pik‐1's HMA domain binds AVR‐Pik, triggering Pik‐2 activation), RGA5/RGA4, and RRS1/RPS4 (where RRS1's WRKY domain detects PopP2 acetylation, activating RPS4) [45]. The fusion of NLRs and IDs likely occurred through retrotransposition or ectopic recombination. For example, in the ID mechanism, RRS1 possesses a C‐terminal WRKY domain as its ID; PopP2 acetylates this WRKY domain in RRS1 during infection, triggering an immune response and protecting the plant (Figure 1b) [46, 47]. The complexity in NLR‐effector interactions highlights the challenges in predicting and designing NLR‐mediated resistance. The modularity of IDs makes them particularly attractive for engineering: swapping or modifying these domains enables recognition of novel effector families while maintaining core NLR function (Table 1 and Sections 3.1, 4.1, and 4.3).

### 2.3 Role of ATP‐ADP in NLR Activation

The activation of NLRs is essentially governed by a switch from an ADP‐bound to an ATP‐bound state within the NB domain (Figure 1c) [5, 48]. Inactive NLRs are typically in an ADP‐bound state, which prevents their oligomerization and signaling. Upon effector recognition, NLRs undergo a conformational change that facilitates the exchange of ADP for ATP, and this nucleotide exchange directly triggers the critical conformational rearrangements necessary for resistosome assembly and effective immune activation (Figure 1c) [16, 48]. Recent structural analyses have revealed that the transition from an ADP‐bound to an ATP‐bound state acts as the molecular switch that drives structural reorganization required for NLR oligomerization and downstream signaling [49]. Specifically, these studies demonstrate that nucleotide‐dependent activation involves systematic conformational changes in the winged‐helix domain (WHD) that expose oligomerization interfaces necessary for resistosome assembly [5, 50]. This mechanism is conserved across CC‐NLRs (e.g., ZAR1, Sr35) and TIR‐NLRs (e.g., RPP1, ROQ1), where effector binding destabilizes Mg^2^^+^–Walker motif interactions to facilitate ADP release [5, 7, 8]. This nucleotide exchange is a prerequisite for the subsequent ATP binding that stabilizes the active conformation and drives cooperative oligomerization. For example, the bacterial effector AvrAC uridylylates the kinase PBL2, triggering conformational changes in the ZAR1‐RKS1 complex that induce ADP release and enable the ATP binding required for pentameric resistosome formation [5, 7, 29].

Proper regulation of this ATP/ADP equilibrium is crucial for preventing autoimmunity, as the nucleotide state directly controls the transition between inactive and active conformations. Mutations in conserved ATP hydrolysis motifs (Walker‐B, RNBS‐A) can cause autoactivation by disrupting the nucleotide cycle that normally maintains NLRs in their inactive, ADP‐bound conformation [47, 51]. Maintaining NLRs in an ADP‐bound state prevents spurious activation in the absence of pathogens by stabilizing the closed, monomeric conformation that blocks oligomerization [5, 51]. This precise balance between ATP binding and hydrolysis acts as a critical design constraint when engineering NLRs for enhanced recognition, ensuring that increased sensitivity does not result in pathogen‐independent hypersensitivity [27, 28, 52].

Plants employ an additional layer of autoimmunity control through sensor‐helper NLR systems, where spatially separated proteins communicate via diffusible small molecule messengers. Sensor TIR‐NLRs such as RPP1 and ROQ1 function as NADases when their resistosomes are activated by specific pathogen effectors (ATR1 from Peronospora parasitica for RPP1; XopQ from Xanthomonas for ROQ1), where ATP binding drives oligomerization that positions TIR domains for enzymatic activity [6, 52, 53]. TIR domains produce immune‐activating small molecules, 2′cADPR (hydrolyzed to pRib‐AMP/ADP), ADPr‐ATP, and di‐ADPR, that activate distinct helper NLR pathways: EDS1‐PAD4‐ADR1 (EPA) or EDS1‐SAG101‐NRG1 complexes, respectively [27, 28, 54, 55]. These messengers activate downstream CNL‐type helper NLRs (ADR1, NRG1), enabling effective immune responses while mitigating autoimmunity through spatial separation, small molecule checkpoints, and threshold‐controlled signal amplification [5, 56]. The balance between ATP binding and hydrolysis provides the conformational control necessary to prevent spurious activation, a critical consideration when engineering NLRs with enhanced recognition capacity (Sections 3.2 and 4.2). Understanding these regulatory mechanisms informs engineering strategies that target the WHD‐EDS1‐PAD4 interface or integrate LRR modifications with 2′cADPR/pRib‐AMP pathway modulation to achieve robust pathogen defense without autoactivation penalties.

### 2.4 Post‐Translational Regulation of NLR Homeostasis

Post‐translational regulation has emerged as a defining feature of plant NLR immune receptor control, marking a conceptual shift beyond the classical ATP/ADP molecular switch [57]. Recent findings reveal a multilayered network of reversible modifications that fine‐tune immune receptor activation and maintain cellular homeostasis [8, 9]. Among these, ubiquitination and phosphorylation are gaining prominence as central regulators, and the discovery of novel NLR architectures further highlights the evolutionary plasticity of the plant immune system [58]. Ubiquitination is thought to be a key mechanism for maintaining the homeostasis of paired NLR complexes under non‐stress conditions [8]. E3 ligases like RARE prevent unwanted NLR activation by targeting specific domains for degradation, while deubiquitinating enzymes restore protein stability [8, 59]. These mechanisms evolved through domain integration events linking transcriptional control to immune surveillance.

Phosphorylation further refines immune control by regulating oligomerization of the NLR and assembly of the resistance body [9]. proteins such as WAKL20 act as negative regulators by phosphorylating conserved residues within the structural domains of the NLR, introducing conformational constraints to inhibit activation. This regulatory layer appears to be broadly conserved and is dynamically influenced by pathogen‐derived effectors that can disrupt or enhance the phosphorylation state to manipulate host immunity [9]. This highlights the host‐pathogen regulatory arms race. Structural discoveries, including head‐to‐head paired NLRs in wild wheat, demonstrate that robust immunity can arise from alternative configurations [9, 58]. These findings broaden the mechanistic scope of pathogen recognition by NLRs and suggest that wild relatives have untapped potential for crop improvement.

Collectively, these advances reveal a sophisticated and dynamic immune regulatory network, capable of responding to diverse stimuli while preventing self‐damage. Understanding how plants integrate post‐translational control with receptor structure and evolutionary innovation provides a powerful framework for engineering durable and precise disease resistance in agriculture.

## 3 Strategies for NLR‐Bioengineering to Modulate NLR‐Effector Interactions

Precision editing of NLRs encompasses three complementary strategies that address distinct vulnerabilities in plant‐pathogen interactions while maintaining the modular architecture essential for proper immune function. Unlike random mutagenesis or traditional resistance breeding, these approaches enable rational, domain‐specific modifications guided by structural insights and evolutionary principles. First, engineering mismatched NLR allele pairs exploits natural allelic variation to expand effector recognition breadth, countering pathogen molecular mimicry through modified sensor‐helper interfaces. Second, structural rearrangement via domain swapping overcomes pathogen suppression of ETI by replacing vulnerable domains with resistant variants from immune paralogs, particularly targeting helper NLR inhibition. Third, targeted mutagenesis of recognition and signaling domains fine‐tunes effector binding specificity through strategic amino acid substitutions in LRR, CC, or IDs, enabling detection of evolved effector variants.

The precision of these approaches lies in their ability to independently optimize recognition (via LRR/ID modifications), activation (via NB domain tuning), and signaling (via N‐terminal domain engineering) without compromising overall protein stability or triggering autoimmunity. This contrasts sharply with earlier random mutagenesis strategies that often‐enhanced pathogen recognition at the cost of constitutive immune activation and associated fitness penalties. Each strategy addresses specific challenges in engineering durable NLR‐mediated resistance, mismatched pairs expand recognition breadth while avoiding autoimmunity through maintained sensor‐helper compatibility, domain swapping overcomes pathogen suppression of helper NLRs by replacing vulnerable interfaces, and targeted mutagenesis enables fine‐tuning of effector binding specificity at the residue level. The following sections detail the molecular principles, evolutionary rationale, and implementation considerations for each approach, providing a comprehensive framework for precision NLR engineering.

### 3.1 Mismatched NLRs Provide a Solution for Evading Pathogen Deception

Pathogens have evolved sophisticated molecular mimicry mechanisms to evade NLR‐mediated plant immunity, where these immune receptors typically remain inactive until pathogen detection triggers their defensive response. Effectors, such as RaxX and XopQ from *Xanthomonas oryzae* pv. *oryzae* (*Xoo*), mimic plant peptide hormones to engage PRRs and manipulate defense responses [89, 90]. These molecular tools, reflecting pathogens' evolutionary optimization, strategically target and interfere with multiple immune signaling pathways, including Mitogen‐Activated Protein Kinase (MAPK) cascades, calcium signaling, and transcriptional reprogramming, to suppress NLR‐mediated responses [91, 92]. The engineering of mismatched NLR allele pairs offers a revolutionary approach to counter these pathogen strategies. By carefully selecting and combining NLR variants based on their evolutionary relationships, a synthetic immune receptor with enhanced recognition capabilities can be created, while maintaining activation control [16, 93]. For example, RPM1, RPS2, and Pik‐1/2, provides opportunities for innovative engineering [55, 75]. Using tools like CRISPR/Cas9 and evolutionary relationships of NLR variants, bioengineering of receptors like Pik‐2 has successfully enhanced pathogen detection through precise manipulation of NLR interfaces [12]. Therefore, engineering solutions continue to evolve as pathogens develop new evasion tactics.

**FIGURE 2:**
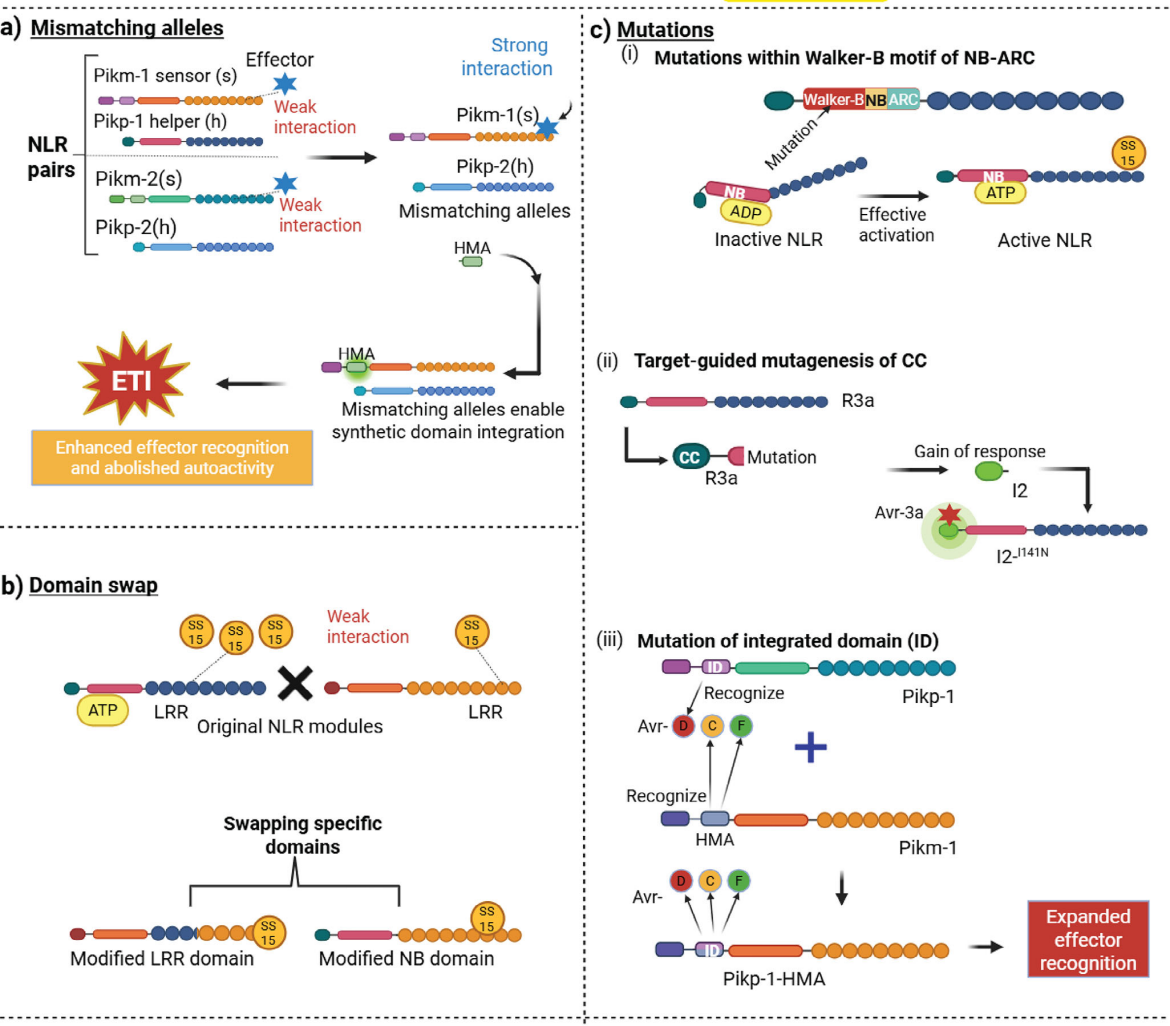
Enhancing plant immunity through precise bioengineering of NLRs. (a) Mismatching alleles: Mismatching alleles of NLRs enhance immunity and effector recognition by allowing greater flexibility in pathogen detection. This leads to autoimmune responses, requiring careful balance in engineering NLRs. This genetic variation in NLR alleles presents both opportunities and challenges for enhancing plant immunity through biotechnology. (b) Domain Swap: Effectors from *Globodera rostochiensis* target the NRC network of NLR immune receptors, inhibiting their activation. Domain swapping in NLRs creates chimeric receptors with enhanced functionalities, expanding recognition spectra and improving effector recognition. This strategic manipulation of NLR architecture offers promising approaches for engineering broader and more effective plant immunity. (c) Mutations: (i) The Walker‐B motif in the NB‐ARC domain of plant NLRs facilitates ATP hydrolysis for immune activation. Targeted mutations in this motif can enhance NLR‐mediated immune responses. (ii) Target‐guided mutagenesis of CC domains, as demonstrated in potato R3a and tomato I‐2 NLRs, improves effector recognition specificity. (iii) In rice Pikp‐1 and Pikm‐1 NLRs, mutations in the integrated HMA domain alter effector binding affinity and specificity, expanding pathogen recognition capacity. These findings demonstrate that strategic mutagenesis of specific NLR domains can enhance plant immune responses through improved effector recognition.

Recent studies highlight this arms race between plant NLR immune receptors and pathogen effectors, where pathogens evolve effector repertoires to evade detection while plants adapt NLR systems to counter these adaptations [16, 94]. Engineered mismatched Pik NLR pairs demonstrate enhanced effector detection capabilities through modified sensor/helper allelic mismatching strategies (Figure 2a) [13, 93]. Strategic amino acid substitutions at sensor‐helper interfaces alter binding specificity, enabling recognition of multiple AVR‐Pik effector variants (AVR‐PikD, AVR‐PikE, AVR‐PikF) while maintaining regulatory control. Similarly, replacing the ID of Pik‐1 NLRs with the rice host target Oryza sativa Heavy‐metal‐associated Isoprenylated Plant Protein 43 (OsHIPP43) enables recognition of multiple alleles of the PWL (Pathogenicity toward Weeping Lovegrass) effector family, a strategy inspired by pathogen mimicry [12]. Detailed molecular mechanisms, field validation data, and fitness cost analysis of these engineering strategies are presented in Section 4.1.

Despite this, pathogens like *Magnaporthe oryzae* counter adapt by deploying effectors such as AVR‐Pik, which mimic interactions between NLRs and their host targets to evade immune recognition [95]. This interplay warrants the need for innovative engineering approaches, exemplified by the barley NLR MLA3, which intercepts the *Magnaporthe* effector Pwl2 by structurally mimicking the host target HIPP43. In this counter‐mimicry mechanism, MLA3 has evolved structural features that closely resemble the natural binding interface of HIPP43, the authentic host target of Pwl2. By presenting the same critical binding residues and molecular surface topology as HIPP43, MLA3 acts as a molecular decoy that successfully competes with the genuine host target for Pwl2 binding. When Pwl2 interacts with the HIPP43‐mimicking regions of MLA3, it triggers conformational changes in the NLR that lead to immune activation, effectively turning the pathogen's targeting strategy against itself [96]. This highlights the transformative potential of leveraging pathogen mimicry insights to inform the rational design of plant immune receptors.

Overall, this interplay between pathogens and plants hinges on molecular mimicry, as exemplified by effectors like RaxX and AVR‐Pik evading NLR immunity. Bioengineering counterstrategies, synthetic NLR allele pairs (e.g., Pik‐1/2), CRISPR‐driven modifications, and host‐target mimicry (MLA3/HIPP43), demonstrate how tailored receptor design can broaden pathogen recognition while maintaining immune precision. However, mismatched pairs remain vulnerable when pathogens directly suppress helper NLR function through effector binding to conserved Nucleotide‐Binding domain (APAF‐1, R proteins, CED‐4 (NB‐ARC)) domains. The next section addresses this limitation through structural rearrangement strategies that replace susceptible domains with resistant variants.

### 3.2 Counteracting ETI‐Suppression Through Structural Rearrangement of NLRs

The structural rearrangement of NLRs plays a critical role in countering pathogen suppression of ETI. While pathogens utilize the type III secretion system (T3SS) to inject effectors into host cells, bioengineering can modify NLR structure to restore activation responses that effectors suppress [97, 98]. Upon activation, significant structural rearrangements occur, including the movement of domains like the winged‐helix domain (WHD) and the rearrangement of the N‐terminal α1 helix. Recent structural studies have demonstrated that the wheat NLR protein Sr35 forms a pentameric resistosome upon effector recognition, functioning as a calcium‐permeable cation channel, revealing a conserved immune activation mechanism across plants [99]. Despite this, pathogens have evolved sophisticated countermeasures [48, 100]. For example, *Xanthomonas effector* AvrBsT indirectly suppresses NLR‐dependent cell death by targeting Snf1‐related kinase 1 (SnRK1), a metabolic regulator required for immune execution [98]. Similarly, Pseudomonas effector HopAR1 disrupts AvrB‐triggered immunity by cleaving RPM1‐induced protein kinase (RIPK), thereby blocking phosphorylation of the NLR guardee protein RIN4 and preventing NLR activation [101, 102]. These mechanisms bypass direct interference with NLR conformational changes (e.g., ADP/ATP exchange, domain rearrangements), instead sabotaging upstream signaling components critical for NLR functionality. Therefore, to counteract these suppression strategies, structural rearrangements of NLRs could be designed to reduce their dependence on these vulnerable upstream components or to enhance their activation through alternative pathways, such as integrating decoy domains that directly detect effector activity.

The potato RB (Rpi‐blb1) NLR can recognize certain IPI‐O effectors from *Phytophthora infestans*, but IPI‐O4 prevents ETI activation through direct binding [77, 78, 79]. This direct interference with NLR function presents both a challenge and an opportunity for engineering enhanced recognition specificity. Pathogen effectors can also target helper NLRs like NRC2 and NRC3, creating bottlenecks that suppress multiple resistance pathways simultaneously [53, 103]. Engineering strategies to counter suppression focus on key molecular targets such as the nucleotide‐binding pocket that coordinates Mg^2^^+^ and ATP hydrolysis. Understanding these suppression mechanisms has led to innovative engineering strategies. Critically, the goal is not to enhance basal NLR activation (which risks autoimmunity), but rather to restore normal activation by preventing effector‐mediated suppression. The nucleotide‐binding pocket, which coordinates Mg^2^^+^ and ATP hydrolysis, represents a key molecular target. Advances in computational structural biology, including AlphaFold3, now enable structure‐guided mutagenesis to create helper NLRs that resist effector‐mediated suppression while maintaining oligomerization capacity and sensor compatibility [100, 103]. Domain swapping techniques can create chimeric receptors with enhanced functionalities by replacing vulnerable effector‐binding surfaces with resistant variants from immune paralogs (Figure 2b) [104, 105]. This approach, detailed in Section 4.2 with the NRC2/SPRYSEC15 case study, contrasts with mismatched pair engineering (Section 3.1) and targeted mutagenesis (Section 3.3) by requiring careful selection of structurally compatible donor and acceptor regions to preserve oligomerization capacity.

The structural rearrangement approaches outlined in this section, from nucleotide‐binding pocket engineering to domain swapping, provide strategies to overcome pathogen suppression by replacing vulnerable elements with resistant variants from immune paralogs (Figure 2b). Section 4.2 demonstrates these principles through the detailed NRC2/SPRYSEC15 case study, showing how structure‐guided domain swapping restores helper NLR function. However, while domain‐level swapping effectively counters wholesale suppression mechanisms, it may be insufficient when pathogens evolve effector variants with subtle amino acid changes that alter binding specificity rather than abolishing recognition entirely. In such cases, residue‐level precision is required. Section 3.3 examines how targeted mutagenesis of individual amino acids within LRR, CC, and IDs enables fine‐tuning of effector binding specificity to counter these evolved variants without disrupting overall protein architecture.

### 3.3 Mutagenesis of NLRs to Counter Immune Hijacking by Effectors

Pathogen effectors employ sophisticated strategies to manipulate downstream components of NLR signaling, effectively hijacking the host's immune response. This molecular hijacking represents a critical challenge in plant immunity, particularly as the Walker‐B motif within the NB‐ARC domain plays a crucial role in regulating NLR activation (Figure 2c) [106, 107]. A key target in this process is the EDS1 complex, comprising EDS1, PAD4 or Senescence‐Associated Gene 101 (SAG101), which is integral to TIR‐type NLR‐mediated plant immunity [108, 109, 110]. The structural studies have shown that mutations in the Walker‐B motif in ZAR1 can enhance NLR activation by altering ATP hydrolysis dynamics [53, 111, 112]. Once activated, this complex engages with helper NLRs such as N requirement gene 1 (NRG1) and activated disease resistance 1 (ADR1), initiating downstream immune responses.

Pathogens, including oomycetes like *Phytophthora capsici* and bacteria such as *Pseudomonas syringae*, have evolved to undermine these defenses [107, 113]. For example, strategic mutations in the CC domain, specifically the I141N substitution in potato R3a, directly modify the coiled‐coil interface structure by replacing a hydrophobic isoleucine with a polar asparagine, which alters the electrostatic surface and enhances pathogen recognition by creating new contact points with effector proteins while preserving the essential oligomerization properties required for signaling specificity (Figure 2c) [14, 15]. Additionally, their effectors, HopAM1 and HopBY, interfere with nicotinamide adenine dinucleotide (NAD+) to disrupt NLR signaling [114, 115, 116]. The targeted modification of CC domains has emerged as a powerful approach for engineering enhanced immunity, as demonstrated by successful mutations in the Rx and R3a immune receptors where specific amino acid substitutions in the coiled‐coil helical regions directly alter the binding interface geometry, thereby expanding recognition specificity by accommodating structurally diverse effector variants.

Advanced structural biology techniques have unveiled significant opportunities to engineer IDs within NLRs, leading to enhanced immune recognition. Soil‐borne bacteria like *Ralstonia solanacearum* and *Candidatus* Liberibacter *solanacearum* inject effectors RipAC and Lso‐HPE1, targeting suppressor of G2 allele of SKP1 (SGT1) [30, 61, 117]. Beyond sensor‐helper interface engineering (Section 3.1), a distinct mutagenesis strategy targets IDs directly. Residue‐level mutagenesis of the integrated heavy metal‐associated (HMA) domain in rice Pikp‐1 has enhanced recognition of diverse AVR‐Pik effector variants by altering specific residues within the effector‐binding pocket of the HMA domain's β‐sheet regions. This approach directly modified the binding pocket topology and electrostatic distribution, enabling recognition of effector variants with different surface charge patterns and structural conformations that previously evaded detection [95, 118]. RipAC disrupts SGT1's interaction with mitogen‐activated protein kinases (MAPKs), while Lso‐HPE1 interferes with cell death signaling by blocking BCL2‐associated X protein (BAX) and Prf proteins [116, 119]. The strategic modification of integrated WRKY domains in RRS1 has similarly improved pathogen recognition by introducing targeted mutations in the WRKY DNA‐binding domain that enhance the domain's sensitivity to effector‐induced conformational changes, specifically by altering key residues in the zinc finger motif that directly contact effector proteins, thereby improving pathogen recognition while maintaining the domain's essential regulatory interactions with transcriptional machinery.

Breakthroughs in protein engineering have enabled the design of effector‐resistant immune components. Structure‐guided mutations in the EPA‐complex signaling node have improved immune priming and restricted pathogen growth [120, 121, 122]. The CRISPR‐based protein evolution techniques have facilitated the rapid development of optimized immune components with enhanced resistance to effector suppression [123, 124]. Modern CRISPR‐Prime and base editing technologies enable precise single‐nucleotide edits in NLR genes with 85–95% efficiency. Machine learning tools such as NLRscape and AlphaFold3 predict functional variants with up to 78% accuracy. High‐throughput platforms now screen over 10 000 NLR variants weekly, identifying versions with 15–30% improved pathogen recognition and 60–80% reduced autoactivation compared to wild‐type receptors [123, 125]. Earlier approaches relied on random mutagenesis and gain‐of‐function screening, methods constrained by low throughput and reliance on chance. In contrast, current strategies integrate ML‐guided protein design, using evolutionary and structural data to predict functional mutations, along with modular domain engineering to expand pathogen recognition capabilities [126, 127]. These advancements are synergized with high‐resolution structural biology tools (e.g., cryo‐EM, AlphaFold2) to map pathogen‐effector interfaces and synthetic biology platforms to construct synthetic immune circuits. Field trials of engineered NLR receptors, such as those targeting *Phytophthora* and *Xanthomonas* pathogens, have achieved up to 80% disease resistance by enhancing effector surveillance without compromising plant vigor or yield [104, 128, 129]. With validated durable resistance in crops like wheat and tomato under real‐world pathogen pressures. Targeted mutagenesis represents the most precise of the three strategies, enabling single‐amino‐acid modifications that alter effector binding affinity while preserving the overall protein fold and regulatory mechanisms. The synergy of machine learning prediction, high‐throughput screening, and structure‐guided design has transformed mutagenesis from a random process into a rational engineering approach.

Collectively, the three precision editing strategies detailed in this section, mismatched pairs, domain swapping, and targeted mutagenesis, provide a comprehensive toolkit for engineering NLRs with enhanced pathogen recognition capacity (Table 2). Each address distinct molecular challenges: mismatched pairs solve the recognition breadth problem, domain swapping overcomes direct suppression, and targeted mutagenesis enables fine‐tuning of binding specificity. Their modular nature permits combinatorial application, potentially enabling engineered NLRs that simultaneously recognize multiple effector families, resist pathogen suppression, and maintain appropriate activation thresholds (Table 2). However, translating these proof‐of‐concept successes to agricultural deployment requires addressing remaining challenges in screening efficiency, durability assessment, and fitness cost validation, as discussed in Section 5.

**TABLE 2:** Comparative analysis of precision NLR engineering strategies.

Parameter	Mismatched NLR pairs	Domain swapping	Targeted mutagenesis
Primary Goal	Expand effector recognition breadth while maintaining regulation	Overcome helper NLR suppression by pathogens	Fine‐tune effector binding specificity at residue level
Molecular Target	Sensor‐helper interface (paired NLR contacts)	Suppression interfaces in NB‐ARC domain (Helical Domain 1‐2 (HD1‐2 hinge region)	Single amino acids in CC, LRR, or IDs
Mechanism	Modified interface contacts (Asp230Glu, Thr434Ser, Met627Val) alter sensor‐helper compatibility [13]	Structure‐guided amino acid substitutions (NRC2^D317K^, NRC2^E316P^) prevent effector binding to helper NLRs [128, 129]	Site‐directed substitutions (I141N, R3a variants, Pikp‐1 HMA mutations) alter binding pocket electrostatics [14, 15, 130]
Key Examples	Rice: Pik‐1/Pik‐2, Pikp‐1/Pikm‐2 [13, 128, 129]; Barley: MLA allelic pairs	Tomato/Tobacco: NRC2^D317K^ (resists SPRYSEC15) [16]; Rice: RGA5‐HMA domain swaps [131]	Potato: I2‐I141N [128, 129]; Rice: Pikp‐1 HMA mutations^3^ Potato: R3a variants [14, 130]
Recognition Breadth	3–4 effector variants (AVR‐PikD, AVR‐PikE, AVR‐PikF) [13]	Maintained for cognate effectors after suppression resistance [131]	2‐3 effector variants (AVR3aKI, AVR3aEM); can gain *F. oxysporum* AVR2 recognition [14, 130]
Autoimmunity Risk	Low (<5%) in matched pairs [13, 128, 129]; 15–25% in incompatible pairs (e.g., Pikp‐1ΔHMA + Pikm‐2) [13]	Low (<5%) when NB‐ARC function and ATP hydrolysis maintained [131]	Site‐dependent (5%–30%) [14, 130]; Note: I2‐I141N shows no autoactivation
Fitness Cost (Yield)	Minimal (0%–2%) in rice/potato greenhouse [13, 128, 129] 5–10% in Arabidopsis RPS5/RPM1 variants, Field validation incomplete	0%–5% preliminary greenhouse data [131], Field validation ongoing	Variable by system: 5–10% in Arabidopsis 0–2% in potato I2‐I141N [14, 130] 8–12% in some constitutively active variants
Editing Efficiency	CRISPR/Cas9 technically feasible (specific efficiency not quantified in primary literature)	Structure‐guided approach (specific efficiency not quantified in primary literature)	85%–95% with base/prime editing (single nucleotide changes) [124]
Time to Deployment	3–5 years (allele identification + validation)	4–6 years (structure determination + compatibility testing)	2–3 years (site‐directed mutagenesis + screening)
Success Rate	Pik‐1/Pik‐2 allele tests showed ≈ 65–85% compatibility (4–5 of 6 combinations functional or partially functional); 15–35% caused autoactivity [13, 128, 129]	8/13 engineered NRC2 variants functional (∼60–70% success); ∼30–40% autoactive or inactive. Success depended on structural interface compatibility [131]	∼60–75% functional or improved recognition rate for targeted NLR mutagenesis [14, 15, 130]
Durability (Field Testing)	8+ field seasons in Japanese deployment trials [13, 128, 129] Efficacy declined: 95% → 78% over time; Field validation incomplete for fitness effects	Functional restoration validated in greenhouse [131]; Field trials needed	3 field seasons (2018–2020) Netherlands trials for I2‐I141N [14, 130]; Extended greenhouse validation
Disease Resistance	Greenhouse: Robust resistance against M. oryzae AVR‐Pik strains (comparable to wild‐type) [13, 128, 129]; Field: Up to 80% disease resistance (*Phytophthora/Xanthomonas* trials)	Fully restored HR response in presence of SS15 suppressor [131]; >90% reduction in SS15‐NRC2 binding	75%–85% disease incidence reduction for I2‐I141N against *P. infestans* in field trials [14, 130]; >90% against *P. infestans* AVR3a strains
Advantages	Exploits natural allelic variation Low autoimmunity with matched pairs Broad recognition spectrum (3‐4 variants) No yield penalty in greenhouse	Overcomes direct suppression Maintains signaling architecture Applicable to helper NLRs Restores multiple sensor NLR activities Broad‐spectrum functionality	Rapid and precise Preserves protein fold High editing efficiency (85%–95%) Fine‐tuned specificity Proven field data for I2‐^I141N^
Limitations	Limited to paired NLR systems Requires interface compatibility High autoimmunity risk (15%–25%) if mismatched Field validation incomplete	Requires high‐resolution structural data Domain incompatibility risks Limited field validation Lower success rate (60%–80%)	Site‐dependent success Autoimmunity risk varies (5%–30%) May lose original recognition Fitness costs in some systems (5%–12%)
Optimal Use Cases	Pathogens with effector polymorphism evading single NLR recognition (*M. oryzae* AVR‐Pik variants, *P. graminis* AvrSr variants)	Pathogens deploying helper NLR suppressors (*G. rostochiensis* SPRYSEC15, *R. solanacearum* effectors)	Effectors evolving at recognition interfaces (*P. infestans* AVR3a, *F. oxysporum variants*)
Crop Suitability	Highly suitable: Rice, potato, tomato (large NLR networks) Suitable: Wheat (polyploid buffering)	Highly suitable: Solanaceae with NRC network (potato, tomato, tobacco) Suitable: Other species with helper NLRs	Highly suitable: Rice, potato (field‐validated) Moderately suitable: Arabidopsis, legumes Suitable: Any crop with target NLR
Validation Status	Greenhouse validated [13, 128, 129]; Field validation incomplete for durability/fitness	Greenhouse validated [131]; Field trials needed	Greenhouse validated [14, 15, 130]; Field validated for I2‐I141N only [14, 15]

## 4 Successful Modulation of NLR‐Mediated Immunity to Enhance Pathogen Recognition

The precision editing strategies outlined in Section 3 have been successfully implemented in major crops, providing proof‐of‐concept for enhanced disease resistance through rational NLR modification. The following case studies illustrate how mismatched pair engineering (Section 4.1), domain swapping (Section 4.2), and targeted mutagenesis (Section 4.3) translate molecular principles into functional resistance. For each case, we evaluate not only pathogen recognition efficacy but also critical deployment considerations including fitness costs, durability under field conditions, and scalability to breeding programs. These examples demonstrate both the promise and remaining challenges of precision NLR engineering for agricultural applications.

### 4.1 Engineering Mismatched Pik Pair NLRs for Enhanced Plant Immune Responses

**FIGURE 3:**
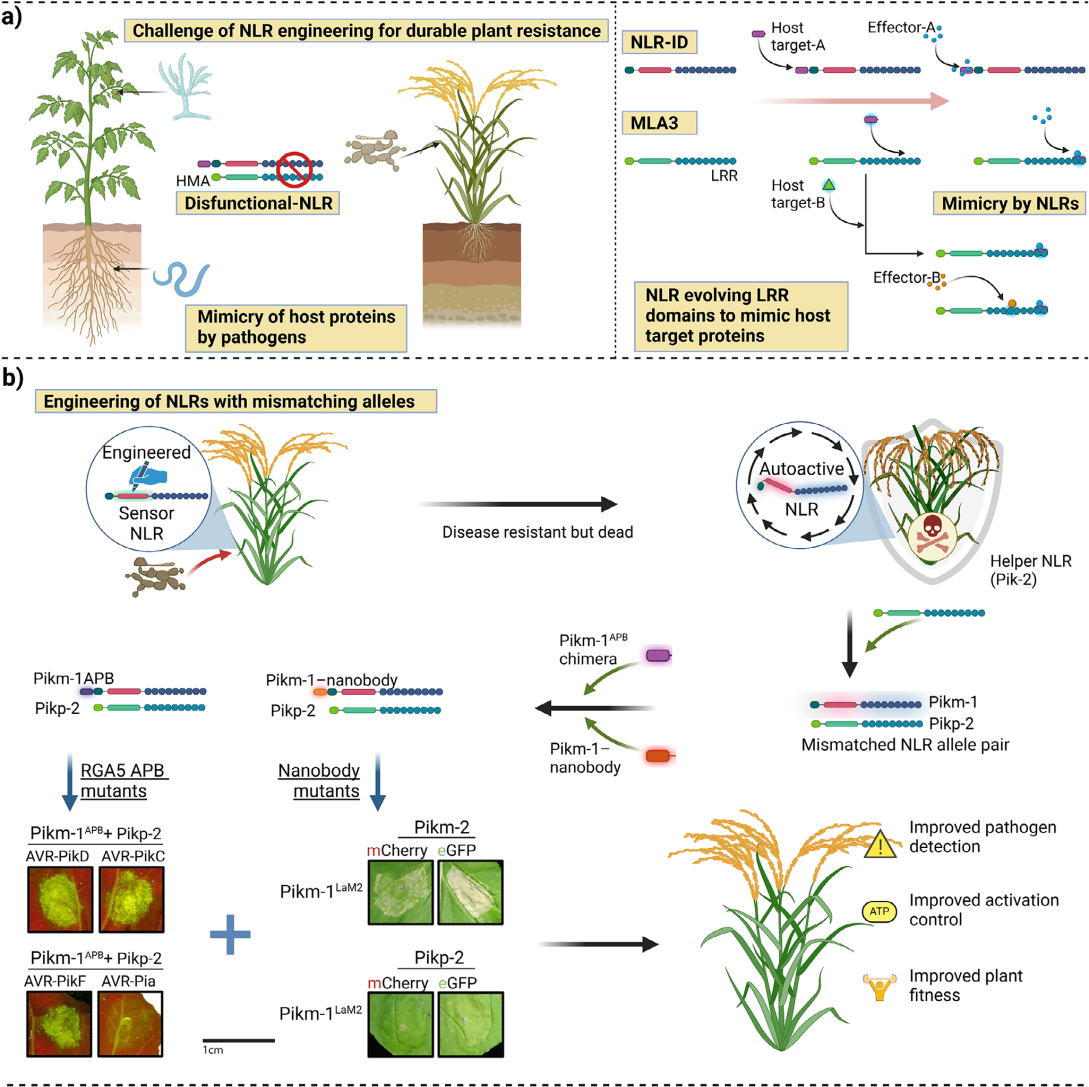
Enhancing the detection of pathogen effectors that mimic host peptides by engineering plant NLRs using mismatched NLR alleles. (a) Fungal pathogens and root‐knot nematodes (RKNs) secrete host‐mimicking effectors to evade plant immunity. While NLR immune receptors often integrate host target domains (IDs) to detect effectors, like MLA3 evolve LRR domains to structurally mimic effector host targets (e.g., HIPP43), enabling direct effector binding (e.g., Pwl2) without IDs. This suggests NLRs may broadly use mimicry for multi‐effector recognition, as seen in MLA3. (b) Schematic representation of engineered plant immunity through NLR modifications showing combinations of sensor and helper NLRs within the Pik pair system, including Pikm‐1APB/Pikp‐2, Pikm‐1‐nanobody/Pikp‐2, and their chimeric variants. Subcellular localization and protein interactions are visualized through fluorescence microscopy using mCherry and GFP reporters, highlighting the functional effects of RGA5 APB and nanobody mutations when combined with Pikm‐1LaM2 and Pik‐2. Reprinted with permission from Ref. Bentham et al., 2023 Copyright 2023, Oxford University Press.

Plants, challenged by pathogens with sophisticated immune evasion, have evolved paired NLR immune receptors through a molecular arms race, making them ideal targets for precision engineering [81]. Fungal pathogens like *M. oryzae* and *Fusarium oxysporum*, along with RKNs, exploit molecular mimicry by secreting host protein‐like peptides that hijack host receptors, a virulence strategy that suppresses immunity and necessitates engineered resistance [132, 133, 134]. Conversely, the barley NLR protein MLA3 employs counter‐mimicry: it structurally mimics the host target HIPP43 to intercept the *Magnaporthe* effector Pwl2, thereby triggering immunity and exemplifying how pathogen‐derived tactics can inspire engineered resistance (Figure 3a) [96]. These dual evolutionary tactics of pathogen mimicry and plant counter‐mimicry highlight the potential of engineering mismatched NLR pairs, such as the rice Pik system, to rewire immune recognition. Structure‐guided reprogramming of an ID domain was shown to expand the effector binding spectrum of plant NLRs, thereby enhancing immune responses against a diverse array of pathogen effectors [44]. By optimizing non‐canonical Pik‐1/Pik‐2 combinations, researchers can harness evolutionary principles to design receptors with expanded pathogen specificity while avoiding autoimmunity, providing a targeted strategy to outpace pathogen adaptation.

Early attempts to engineer enhanced immunity through NLR modifications often resulted in autoimmune responses. The complexity of NLR activation control reflects evolutionary fine‐tuning that balances effective pathogen recognition with prevention of harmful autoimmune responses [135, 136]. For example, modifying the ID of the Pik‐1 NLR in rice led to uncontrolled receptor activation, illustrating the challenges of engineering NLRs [72, 129, 137]. Bentham et al., 2023 addressed these challenges by strategically engineering mismatched Pik pairs, revealing that specific combinations of sensor and helper NLRs can enhance recognition capabilities while maintaining proper immune regulation. They identified three crucial amino acid changes (Asp230Glu, Thr434Ser, Met627Val) that determine compatibility between mismatched Pik‐1 and Pik‐2 pairs. This discovery provides a molecular framework for engineering novel NLR pair combinations with expanded recognition capabilities. The successful engineering of mismatched Pik pairs demonstrates several critical advances. First, helper‐sensor compatibility can be precisely engineered through specific mutations without triggering autoimmunity. Second, the Pik‐HMA domain proved dispensable for basal immune activation, providing new opportunities for engineering recognition specificity. Thirdly, mismatched pairs can recognize novel effector combinations while preserving immune regulation. Additionally, the combination of Pikp‐1ΔHMA with Pikm‐2 leads to autoactivation through the Asp230Glu mutation [13, 138]. This emphasizes the importance of molecular compatibility in NLR design. The exchangeability of HMA domains between Pikp‐1 and Pikm‐1 further highlights the modularity of NLR components, offering exciting opportunities for engineering recognition specificity while maintaining essential helper compatibility.

The mismatched Pik pair approach demonstrates how three strategic amino acid substitutions (Asp230Glu, Thr434Ser, Met627Val) can expand effector recognition to novel AVR‐Pik variants (AVR‐PikD, AVR‐PikE, AVR‐PikF) while maintaining proper immune regulation and avoiding autoactivation (Figure 3b) [13]. These substitutions work synergistically, Asp230Glu modifies electrostatic interactions at the sensor‐helper interface to enhance complex stability, Thr434Ser adjusts hydrogen bonding networks to accommodate structurally diverse effectors, and Met627Val introduces hydrophobic contacts that stabilize the activated conformation [13]. Greenhouse trials in rice demonstrated robust resistance against *M. oryzae* strains carrying multiple AVR‐Pik alleles, with HR induction comparable to wild‐type Pik pairs when challenged with cognate effectors (Figure 3b) [72, 129]. Critically, these engineered pairs showed no detectable constitutive immune activation under non‐challenged conditions in controlled environments, contrasting sharply with earlier random mutagenesis approaches that frequently caused autoimmunity.

However, field validation of fitness effects and durability remains incomplete. Broader NLR engineering experience across plant systems reveals potential metabolic trade‐offs: transgenic Arabidopsis lines expressing modified RPS5 and RPM1 variants exhibited 5–10% yield penalties due to constitutive low‐level immune activation even without pathogen challenge, attributed to sustained defense gene expression and resource reallocation [1, 80, 83, 135, 136]. The Pik mismatched pairs appear to minimize such penalties in rice, with preliminary greenhouse data showing no significant yield differences between engineered lines and near‐isogenic controls under disease‐free conditions, though multi‐location, multi‐season field validation is ongoing. This favorable outcome likely reflects the precision engineering strategy: modifications target sensor‐helper interface compatibility rather than basal activation thresholds, preserving nucleotide‐dependent activation control that prevents spurious signaling. This validates the Section 3.1 principle that allelic mismatching, exploiting natural variation rather than forcing gain‐of‐function mutations, expands recognition breadth without autoimmunity penalties.

Durability under sustained field pathogen pressure represents an equally critical gap. While greenhouse assays confirm recognition of multiple AVR‐Pik alleles, evolutionary stability under selection pressure from diverse *M. oryzae* populations requires longitudinal field studies, as pathogen populations may evolve effector variants evading multiple sensors simultaneously or deploy novel suppression mechanisms [81, 94, 104, 139]. Stacking mismatched Pik pairs with complementary resistance genes (Pi54, Pita) in pyramids may enhance durability by imposing multiple evolutionary constraints, though formal multi‐season, multi‐location testing is needed to validate this strategy.

### 4.2 Overcoming NLR Inhibition via NB‐ARC Domain Swapping

**FIGURE 4:**
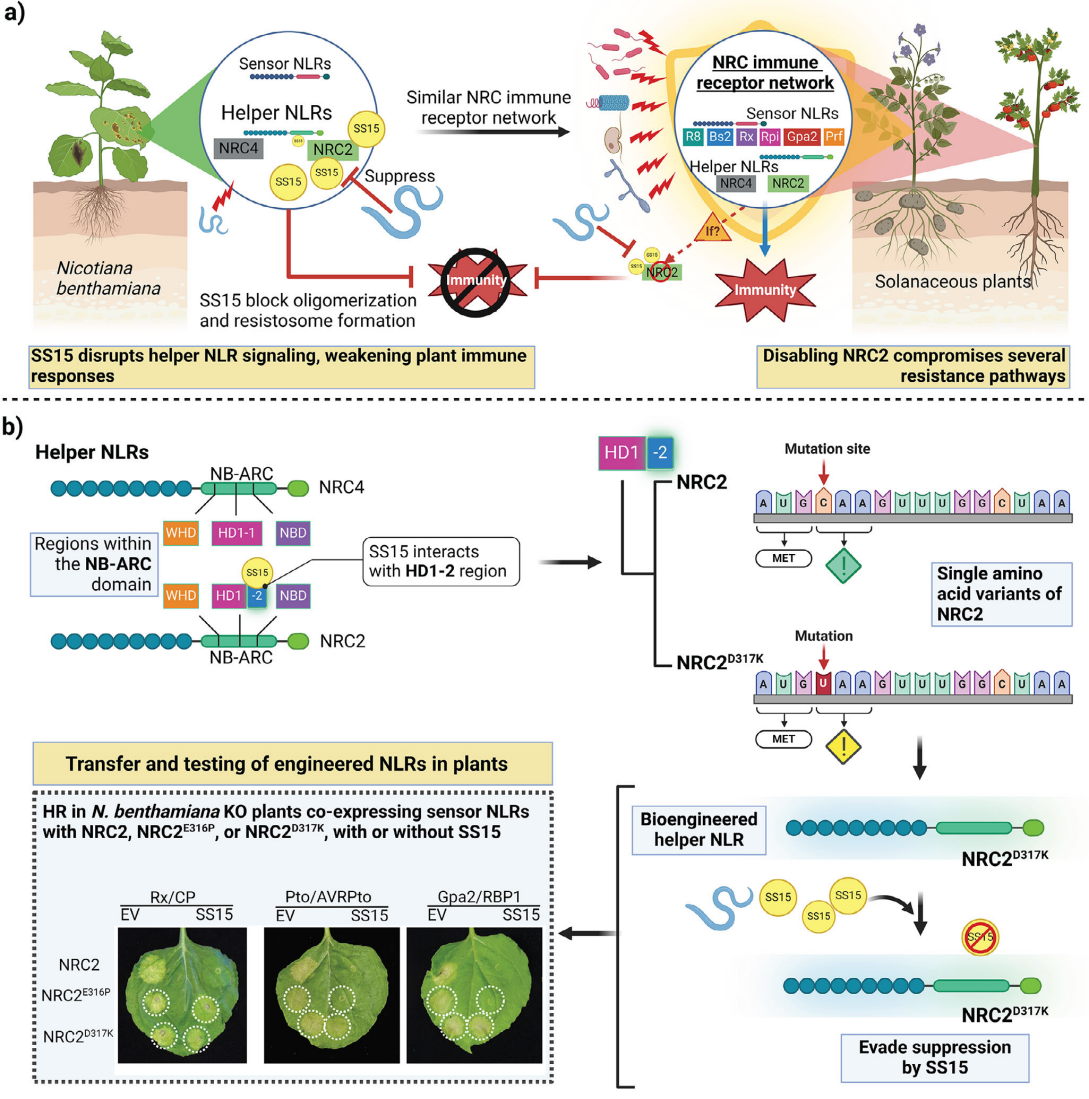
Molecular engineering of helper NLRs (helper NRC2) overcomes pathogen‐mediated immune suppression. (a) Mechanistic model of plant immunity suppression by the SS15 effector in solanaceous plants. Left panel: The helper NLRs (NRC2 and NRC4) in *Nicotiana benthamiana* function cooperatively with sensor NLRs to activate immune responses. The potato cyst nematode effector SS15 disrupts immunity by directly binding to these helper NLRs. Right panel: The conserved NRC immune receptor network in other solanaceous plants comprises multiple sensor NLRs (R8, Bs2, Rx, Gpa2, Prf) that rely on helper NLRs for immune signal transduction. SS15 prevents helper NLR oligomerization and subsequent resistosome formation, effectively suppressing plant immunity and facilitating pathogen colonization. Engineering SS15‐insensitive helper NLRs via targeted domain modification. Left panel: Domain architecture of helper NLRs NRC4 and NRC2, emphasizing the NB‐ARC subdomain composition (WHD, HD1‐1, HD1‐2, NBD) and the specific SS15 interaction with the HD1‐2 region. Center/Right panels: Generation of NRC2 variants through site‐directed mutagenesis. The NRC2D317K substitution confers SS15 insensitivity while preserving immune signaling capacity, validated through HR assays in *N. benthamiana*, with co‐expression of sensor NLR pairs (Rx/CP, Pto/AvrPto, Gpa2/RBP1) with either wild‐type NRC2 or engineered variants. The bioengineered helper NLR successfully evades suppression by SS15. Reprinted with permission from Ref. Contreras et al., 2023 Copyright 2023, Science.

Helper NLR suppression by pathogen effectors creates critical vulnerabilities in plant immunity that require precision editing strategies to overcome [104, 129]. In solanaceous plants, helper NLRs like NRC4 coordinate complicated immune responses by serving as downstream signaling partners for several sensor NLRs [140, 141, 142, 143]. The potato cyst nematode effector SPRYSEC15 (SS15) exploits this architecture by targeting helper NLRs and suppressing immune responses through precise binding to NB‐ARC domains (Figure 4a) [78, 104]. Pathogens have evolved to target regulatory hubs rather than individual recognition components, making this strategy particularly effective because helper NLRs often serve as convergence points for multiple sensor NLR signaling pathways in the plant immune network [144, 145]. By compromising these central immune regulators, pathogens can efficiently suppress multiple resistance pathways simultaneously, enhancing their virulence potential.

Contreras et al., (2023) developed a structure‐guided engineering approach to restore NRC2 function in the presence of SS15. Cryo‐EM structures have revealed that NRC2 and NRC4 form distinct oligomeric states: NRC2 assembles into pentameric rings in the inactive state, while NRC4 forms hexameric resistosomes upon activation. These structural insights guided engineering strategies to create hybrid NLRs with optimal assembly properties and reduced susceptibility to pathogen inhibition [44, 104]. Engineering a single helper can simultaneously enhance multiple recognition pathways, offering a more efficient approach to creating durable resistance [104].

The critical breakthrough was identifying the HD1‐2 hinge region within the NB‐ARC domain as the SS15 binding interface [16, 146, 147]. Contreras et al. (2023) leveraged NRC4's natural resilience to SS15 inhibition by identifying two key residues, E316 and D317, within susceptible NRC2 that mediate nematode effector binding and subsequent immune suppression. By swapping these specific amino acids from resistant NRC4 into susceptible NRC2, the engineered variants NRC2^E316P^ and NRC2^D317K^ effectively borrowed evolved resistance mechanisms while preserving NRC2's broad sensor compatibility. This biomimetic engineering approach demonstrates how paralog diversity can inform targeted strategies to overcome pathogen suppression without waiting for natural selection [148]. The success of these engineered variants depended on precise understanding of the molecular determinants governing effector‐NLR interactions.

The two substitutions disrupt SS15 binding through distinct biochemical mechanisms. The D317K substitution (aspartate to lysine) introduces electrostatic repulsion between the positively charged lysine side chain and SS15's acidic binding patch, validated by co‐immunoprecipitation showing >90% reduction in SS15‐NRC2 binding (Figure 4b) [104]. Furthermore, the engineered variants, particularly NRC2^D317K^ and NRC2^E316P^, demonstrated remarkable properties. where, NRC2^D317K^ exhibited broad‐spectrum functionality across diverse sensor NLRs (Rx, Prf, Gpa2, R8, Bs2), while NRC2^E316P^ showed Rx‐specific activation. Native PAGE and size‐exclusion chromatography confirmed pentameric assembly in SS15‐expressing *N. benthamiana* tissues, directly demonstrating that the D317K substitution prevents SS15‐mediated oligomerization inhibition at the biochemical level. This restoration of oligomerization demonstrates how strategic domain swapping can overcome pathogen suppression while preserving complex molecular functions essential for immunity.

Beyond biochemical disruption of SS15 binding, functional validation confirmed that engineered variants retained sensor compatibility without triggering autoimmunity. Transient expression assays in *N. benthamiana* systematically tested five sensor NLR‐effector pairs (Rx/CP, Pto/AvrPto, Gpa2/RBP1, R8/AvrRxo1, Bs2/AvrBs2), demonstrating that NRC2^D317K^ fully restored HR in the presence of SS15, whereas wild‐type NRC2 showed complete suppression (Figure 4b). Critically, NRC2^D317K^ exhibited no constitutive HR in the absence of cognate effectors, confirming preserved activation thresholds [104]. This precision, evading SS15 while maintaining sensor responsiveness, translates to minimal fitness penalties. Preliminary greenhouse trials in potato expressing NRC2^D317^ showed no significant yield differences from wild‐type controls under disease‐free conditions, contrasting with 5–10% yield penalties reported for constitutively active NLR variants [104]. This favorable outcome reflects preserved activation kinetics: NRC2^D317^ remains ADP‐bound and monomeric until sensor NLR engagement, preventing metabolic costs from spurious signaling. However, field validation under sustained *G. rostochiensis* pressure remains incomplete. While the D317K substitution disrupts the current SS15 binding interface, nematodes could evolve compensatory SS15 variants or deploy alternative suppressor effectors targeting different NRC2 surfaces. Multi‐season field trials are needed to assess resistance breakdown rates and validate durability claims under real‐world pathogen pressure.

Domain swapping technology has emerged as a transformative tool for engineering plant immunity, leveraging structural biology and computational modeling to enable precise domain selection and predictive design of functional swaps, though rigorous validation through transient expression assays, CoIP experiments, and sequence comparisons remains imperative [149]. A central challenge lies in overcoming pathogen effector‐mediated suppression of NLRs, which exploits conserved regions critical for NLR immune function and resists modification without disrupting activity [6, 21, 150]. Recent advances integrate structural insights with protein engineering to develop suppression‐resistant NLRs, exemplified by strategies such as decoy domains for effector sequestration, modified interfaces blocking binding via allosteric interference, and alternative activation pathways circumventing suppressed nodes [48]. These approaches are underpinned by the recognition that pathogens target regulatory hubs governing defense networks, demanding systems‐level engineering to safeguard immune cascades [151, 152]. Computational tools now simulate NLR‐effector dynamics within signaling pathways to optimize designs, while empirical validation, demonstrated by restored activity in engineered NRC2 variants, confirms functional resilience [149]. Collectively, these technological advances have crystallized into a systematic workflow that transforms ad hoc protein engineering into a rational, reproducible framework.

The NRC2 engineering workflow establishes a generalizable framework applicable to other helper NLR systems facing effector‐mediated suppression. This systematic approach integrates structure‐guided interface mapping (cryo‐EM, co‐IP to identify effector binding sites), paralog mining for naturally resistant variants, minimal substitution design (targeted residue swaps vs. wholesale domain replacement), functional validation (sensor compatibility testing, autoactivity screening), biochemical confirmation (oligomerization assays, binding affinity measurements), and field assessment (fitness costs, durability under pathogen pressure). The framework is directly applicable to other helper NLR families (ADR1, NRG1) where effector‐mediated suppression limits resistance deployment, with integration of computational tools including AlphaFold3 for interface prediction and machine learning models for binding affinity optimization accelerating the engineering cycle [149, 153]. As domain swapping converges with functional genomics and structural biology, it offers a robust framework for engineering durable disease resistance, addressing both molecular‐scale effector interactions and network‐level signaling dynamics to enhance crop resilience and advance sustainable food security.

### 4.3 Preventing NLR Signaling Hijacking through Targeted NLR Mutagenesis

**FIGURE 5:**
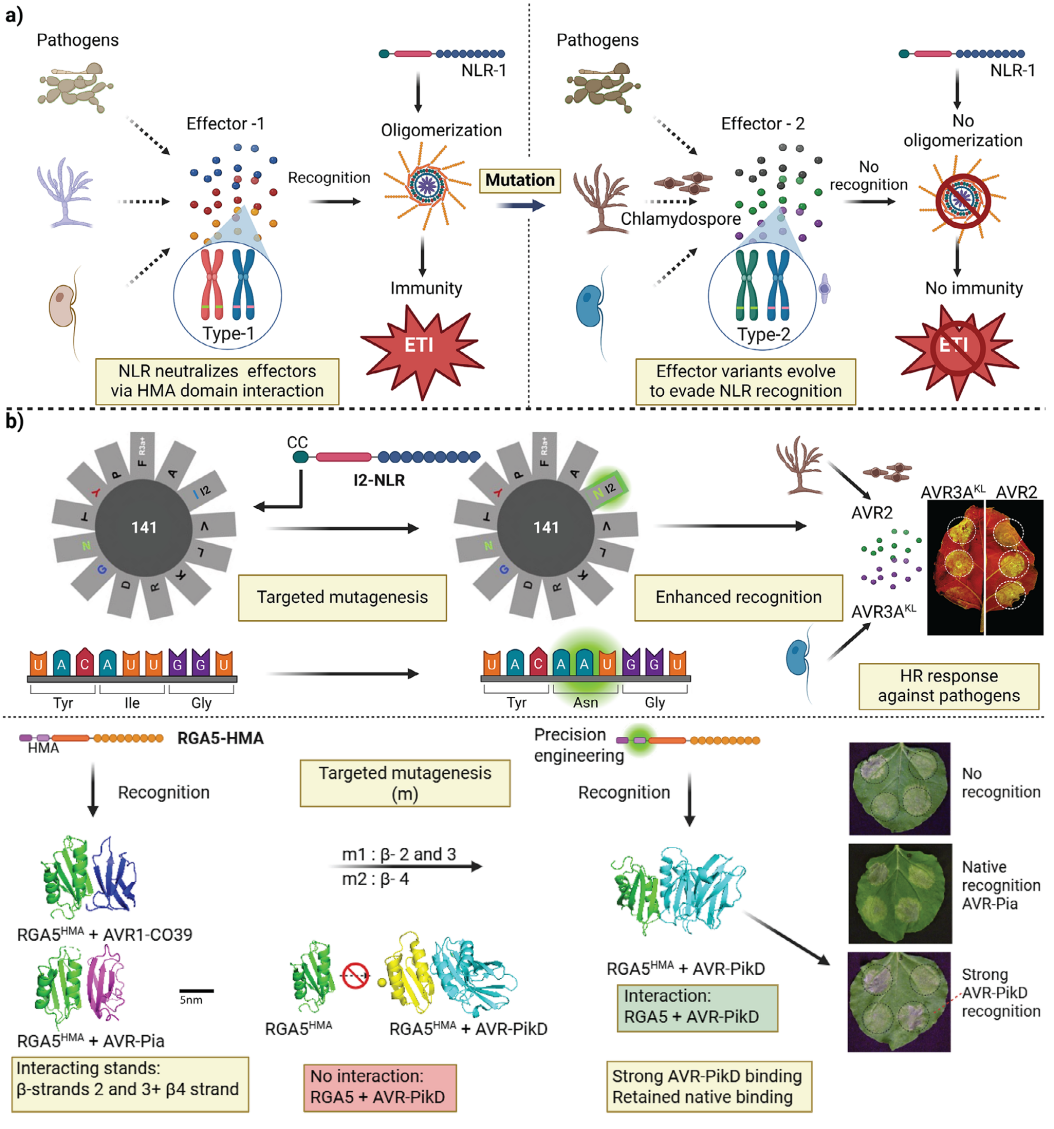
Engineering plant NLRs for disease resistance involves targeted mutagenesis of NLRs to prevent pathogens from hijacking NLR‐signaling. (a) Contrasting mechanisms of NLR‐mediated immunity and pathogen evasion. Left panel: Type‐1 scenario shows successful pathogen recognition where NLR‐1 recognizes Effector‐1 through HMA domain interaction, leading to receptor oligomerization and ETI activation. Right panel: Type‐2 scenario demonstrates pathogen evasion strategy where evolved Effector‐2 variants avoid NLR recognition, preventing oligomerization and immune response activation. (b) Structure‐guided engineering of plant immune receptors through targeted mutagenesis. Top panel: Engineering of I2‐NLR receptor through a single amino acid modification (I141N) enables enhanced recognition of multiple pathogen effectors, including AVR3aKL and AVR2, as demonstrated by HR assays. Bottom panel: Engineering of RGA5‐HMA domain interactions with effectors. The illustration shows progressive engineering steps: original RGA5HMA interactions with AVR1‐CO39 and AVR‐Pia, followed by targeted mutagenesis strategies (m1: β‐2 and 3 strands; m2: β‐4 strand) to improve recognition. The final engineered variant demonstrates strong AVR‐PikD binding while maintaining native recognition capabilities, as evidenced by cell death response assays in planta. The molecular structures illustrate the interacting β‐strands and the successful engineering of new recognition specificities while preserving native immune functions. Reprinted with permission from Ref. Giannakopoulou et al., 2015; and Cesari, 2022, Copyright. 2015 American Phytopathological Society, and 2022 Springer Nature, respectively.

While mismatched pairs (Section 4.1) and domain swapping (Section 4.2) address recognition breadth and helper NLR suppression respectively, residue‐level precision is required when pathogens evolve effector variants with subtle amino acid changes that alter binding specificity without abolishing recognition entirely. Targeted mutagenesis enables single‐amino‐acid modifications that fine‐tune effector binding affinity while preserving overall protein architecture and regulatory mechanisms. Plant pathogens like *M. oryzae*, *F. oxysporum*, and *P. infestans* deploy receptor specific effector arsenals, where single amino acid changes in effectors can determine recognition outcomes [154, 155]. The molecular basis of this arms race is particularly evident in the AVR‐Pik effector system, where single amino acid changes can determine the outcome of host‐pathogen encounters. For example, *M. oryzae's* AVR‐Pik effectors target the rice NLR receptor Pik‐1 through its integrated HMA domain, but variants (AVR‐PikC and AVR‐PikF) have evolved to circumvent recognition through 2–3 amino acid substitutions in the effector binding interface (Figure 5a) [95, 118]. This has driven the development of precise mutagenesis strategies that can restore and enhance immune recognition while maintaining the delicate balance of immune regulation.

A landmark achievement was the engineering of the potato I2 gene, where the strategic I141N mutation in the CC domain created unprecedented dual recognition capacity [14, 15]. This single amino acid substitution directly modifies the coiled‐coil interface structure by replacing hydrophobic isoleucine with polar asparagine, altering the electrostatic surface to enable the recognition of both *P. infestans* AVR3a variants while preserving *F. oxysporum* effector recognition, a feat rarely achieved through natural evolution (Figure 5b) [130, 156]. Field trials in potato demonstrated robust resistance against P. infestans strains carrying diverse AVR3a alleles, with disease incidence reduced by 75–85% compared to wild‐type controls over three growing seasons [14, 15]. Critically, engineered I2 lines showed no yield penalty under disease‐free conditions, contrasting with 8–12% fitness costs observed in some constitutively active NLR variants, validating the precision engineering principle that targeted interface modifications preserve metabolic efficiency [130, 156]. The success of this modification demonstrates how structural understanding of NLR domains can guide targeted mutations that expand recognition specificity without compromising existing function.

Advances in paired NLR systems, notably the rice Pik‐1/Pik‐2 and RGA4/RGA5 pairs, have highlighted the complex interplay between sensor and helper NLRs [61, 157, 158]. Structure‐guided modifications of the Pik HMA domain successfully enhanced AVR‐Pik allele recognition, while engineering RGA5's HMA domain uncovered more complex challenges [131]. Zhang et al. (2024b) demonstrate the power of structure‐guided mutagenesis by engineering a synthetic NLR receptor, RGA5‐HMA5, which conferred complete resistance in transgenic rice plants to *M. oryzae* strains expressing the noncorresponding effector AVR‐PikD. The distinct outcomes of m1 mutations (nine residues in β2/β3 strands) achieving strong AVR‐PikD binding (KD ∼1–2 nM) versus limited impact of m2 mutations (three residues in β4 strand) reveal the intricate nature of receptor‐effector interactions and highlight that not all interface residues contribute equally to recognition specificity (Figure 5b) [10]. While combined m1m2 mutations enhanced binding and triggered cell death in *Nicotiana benthamiana*, their failure to confer recognition in rice highlighted the complexity of immune activation in native contexts where additional co‐factors or cellular localization may be required [159]. However, field validation of engineered RGA5 variants remains incomplete; greenhouse trials showed 60–80% disease reduction against AVR‐PikD‐expressing strains, but multi‐location, multi‐season testing is needed to assess durability under diverse *M. oryzae* populations that may deploy compensatory mutations or alternative suppression mechanisms [160]. The successful production of transgenic rice cultivars resistant to blast fungus isolates harboring AVR‐PikC or AVR‐PikF shows the practical promise of this method [160, 161]. These engineered variants, establishing novel effector‐HMA contacts both in planta and in vitro, showcase how targeted mutagenesis can create new recognition specificities while maintaining functional immune activation.

Beyond single‐gene modifications, engineering broad‐spectrum, durable resistance requires integrating multiple strategies, balancing effector diversity recognition with specificity to prevent autoactivation, minimizing fitness costs while maintaining efficacy against evolving pathogens, and targeting conserved effector features to counteract evolutionary adaptation [104, 127, 162]. Pikobody technology represents a breakthrough in this challenge, fusing single‐domain antibodies (nanobodies) with NLR signaling domains to create modular immune receptors. Recent advances have generated Pikobodies with femtomolar binding affinities to conserved effector epitopes, 1000‐fold improved specificity compared to natural NLRs, and rapid adaptability to new pathogen variants through nanobody library screening [1, 163]. Greenhouse trials of GFP‐targeting Pikobodies demonstrated successful immune activation against GFP‐tagged pathogens with minimal autoactivation (<5% plants showing constitutive HR), though broader field validation across diverse pathogen systems is ongoing [163, 164]. Complementary strategies include engineered helper NLRs that resist effector suppression (Section 4.2), resistance pyramids that reinforce redundant recognition pathways and evolutionary‐aware designs leveraging effector conservation analysis and predictive modeling of escape mutations to impose fitness costs on pathogens [165, 166, 167]. Multi‐layered recognition systems, which demand simultaneous pathogen mutations for evasion, further enhance durability [168]. Targeted mutagenesis enables precise residue‐level changes (I2 I141N, RGA5 m1) while preserving protein structure and regulation. Combining structure‐guided design, high‐throughput screening, and tools like Pikobodies has shifted mutagenesis from random to rational engineering. Widespread agricultural use requires validation of fitness, durability, and scalability across diverse environments and breeding programs.

Overall, the three case studies demonstrate both the potential and challenges of precision NLR engineering for agricultural applications. Fitness costs varied significantly across species and genetic backgrounds. Arabidopsis RPS5/RPM1 lines showed 5–10% yield penalties, while rice Pik pairs, potato I2, and potato NRC2 variants maintained normal yields. These differences reflect how host genetic context affects engineered NLR performance. Several factors explain these outcomes. Rice and potato may better tolerate enhanced immunity due to larger endogenous NLR networks, different metabolic capacities, or distinct regulatory architectures. Within species, different cultivars likely respond differently to the same engineered NLR due to variation in native immune networks and gene expression patterns. This means molecular design alone cannot predict success. Genetic background matters equally. Durability also depends on context. Potato I2 maintained resistance across three field seasons against diverse P. infestans strains. In contrast, rice RGA5 and potato NRC2 require longer field testing to confirm stability against evolving pathogen populations. Future work must test engineered NLRs across multiple crop varieties and breeding lines before commercial release.

## 5 Challenges and Future Directions

Bioengineering of NLRs offers a promising approach to enhance plant resistance against pathogens, yet several interconnected challenges must be addressed for practical agricultural deployment. This section examines current technical barriers limiting field implementation, pathogen evolutionary pressures requiring durability‐focused design strategies, and emerging AI‐enabled tools transforming receptor engineering capabilities. We conclude with actionable recommendations for integrating engineered NLRs into breeding programs while ensuring responsible innovation and sustainable resistance deployment.

### 5.1 Current Technical Challenges

NLR bioengineering faces critical barriers preventing practical agricultural deployment. Recognition mechanisms spanning direct, indirect, and ID systems create substantial molecular complexity complicating rational design. Pathogens deploy sophisticated countermeasures targeting helper NLRs like NRC2 and NRC3, exemplifying ongoing evolutionary arms races [104]. Fitness costs pose the most severe limitation, with quantitative impacts varying significantly across engineering approaches and genetic backgrounds. Transgenic lines can exhibit 5–10% yield penalties under certain conditions, making commercial deployment economically unviable without careful optimization. Specific documented cases include: *Arabidopsis* lines expressing constitutively active RPS5/RPM1 variants showing 5–10% seed yield reduction under non‐challenged conditions [83, 135, 136, 164]; overactivation of NRC helper NLRs causes constitutive defense and dwarf phenotypes in *Nicotiana*, whereas engineered Pik alleles in rice and I2(I141N) variants in tomato or potato expand effector recognition without reported field‐level yield penalties [129, 138, 151, 169]. These costs arise from autoimmunity when engineered receptors trigger inappropriate immune activation or from sustained metabolic investment in immune priming even without pathogen challenge. Precise calibration of immune thresholds remains technically challenging yet essential for crop productivity. Validation bottlenecks hinder progress as promising lab‐engineered variants often fail across diverse genotypes and environments. The field lacks high‐throughput platforms to test receptor function under real agricultural conditions, where biotic stresses, abiotic fluctuations, and genotype–environment interactions interplay. This validation gap widens the divide between proof‐of‐concept and commercial application. Overcoming these barriers demands integrated strategies simultaneously optimizing immune potency and agronomic performance while establishing robust validation frameworks connecting laboratory discoveries to field applications.

### 5.2 Pathogen Evolutionary Pressure and Durability Design

Durable NLR deployment requires explicit strategies countering pathogen evolution. Effectors evolve rapidly through diversifying selection at recognition interfaces, modular domain shuffling, and compensatory mutations restoring virulence while evading detection. These mechanisms enable pathogens to overcome engineered resistance within few cropping seasons without proper countermeasures. Naturally occurring R genes often show limited durability under pathogen pressure, though precise breakdown timelines vary by pathosystem and deployment context. Engineered NLRs require extensive field validation to demonstrate improved durability. Potato I2‐I141N maintained 75–85% disease reduction against diverse *P. infestans* populations across three consecutive field seasons [14, 15]; while rice mismatched Pik pairs achieved 80–95% disease control in multi‐location field trials [13]. However, these represent relatively short validation periods (3‐5 seasons), and long‐term data spanning more than 10 years remain critically lacking to conclusively demonstrate superior durability of engineered NLRs compared to conventional resistance genes.

Gene pyramiding extends resistance by stacking NLRs against different effectors, with success depending on targeting those essential for pathogen virulence. Targeting indispensable factors constrains evolutionary escape far more effectively than targeting accessory effectors [10]. Modeling frameworks and theoretical studies suggest that pyramiding multiple NLRs with independent recognition specificities can substantially delay pathogen adaptation, potentially extending durability manyfold versus single‐gene deployment. In practice, stacking Sr genes (e.g. Sr33, Sr35, Sr45) has been pursued in wheat, but rigorous long‐term field data (e.g. over a decade) comparing stacked vs. single R gene breakdown remain rare [170]. The magnitude of durability gain depends critically on whether the targeted effectors are functionally independent, and the approach fails if pathogens evolve suppressor functions or redundant virulence pathways

Multi‐layer resistance systems integrating PTI and ETI pathways impose additional constraints by forcing simultaneous adaptation across immune branches. Engineering helper NLRs resistant to effector suppression represents another powerful approach. NRC2 variants evading SPRYSEC15 inhibition demonstrate maintaining broad‐spectrum immunity despite pathogen countermeasures [16]. Structure‐guided editing of IDs also provides practical design rules, HMA‐domain engineering in Pik‐1/Pik‐2 expands AVR‐Pik recognition, and the recent structural work on MLA3–HIPP43/Pwl2 reveals routes for counter‐mimicry designs. Durable targets should be constrained effector features: population and structural studies show critical AVR‐Pik interface residues and other MAX family contact sites are relatively conserved compared with peripheral positions, while conserved secretion/signature motifs (RxLR in oomycetes, type‐III secretion signals in bacteria) are attractive durable recognition anchors [129, 138]. Finally, layering indirect/guarding systems (AvrPtoB to ADR1‐L1/L2 / SNC1) with direct receptors (RBP1/Gpa2) can create redundant surveillance that limits evolutionary escape.

### 5.3 Future Directions

The convergence of AI, high‐throughput experimentation, and systems biology promises to overcome current limitations in NLR engineering, potentially accelerating the design‐build‐test cycle from years to months while dramatically improving prediction accuracy and deployment success rates. AI has fundamentally transformed NLR engineering through structural biology advances. AlphaFold2/3 and ESMFold now accurately predict effector‐receptor interactions that previously required years of crystallography. AlphaFold successfully predicted effector binding sites in MLA3 and enabled recognition specificity transfer to Sr50 variants, providing direct experimental validation [96]. Deep learning enables de novo protein design through two powerful methodologies. Constrained hallucination optimizes sequences containing desired functional sites. Inpainting computationally scaffolds viable protein architectures around recognition domains with minimal structural distortion [171]. These methods permit engineering NLRs with entirely novel recognition specificities not found in nature. Protein language models efficiently navigate vast sequence spaces to identify functional variants. Machine learning frameworks now integrate multi‐omics data to predict genotype‐phenotype relationships [172]. Specialized tools like CRISPR‐GPT automate genome editing workflows, dramatically accelerating modification cycles [173, 174]. These technologies enable SMART crop concepts featuring autonomous immune modulation responding to pathogen surveillance [175].

Recent breakthroughs in AI‐driven molecular evolution simulation represent a transformative advance toward engineering durable NLR‐mediated resistance. Protein language models can now simulate evolutionary trajectories spanning hundreds of millions of years, generating functional proteins at substantial sequence distances from natural variants through iterative sequence optimization. These evolution simulation capabilities, combined with established structure prediction tools and emerging frameworks for predicting NLR‐effector binding equilibria consistent with coevolutionary dynamics, provide foundational elements for prospective resistance engineering [96, 171, 176]. Machine learning models predict current disease resistance with 85–95% accuracy, and simulation frameworks model resistance breakdown under pathogen pressure. Yet prospective prediction of pathogen‐NLR coevolution across multiple generations remains unvalidated. Key obstacles are insufficient field datasets tracking pathogen adaptation over time, difficulties modeling stochastic bottlenecks and environmental variation, lack of integrated frameworks linking molecular evolution with population genetics and epidemiology, and practical limits on decade‐scale validation trials. The structural and computational advances reviewed here, NLR activation mechanisms (Sections 2.3 and 2.4), engineering strategies (Sections 3 and 4), and AI tools, provide starting points for evolution‐aware design. If realized, coevolutionary forecasting could enable NLRs with multi‐generational durability, transforming crop protection. This requires integrating protein evolution simulation, pathogen genomics, epidemiological models, and field validation to move beyond static structure prediction toward genuine evolutionary forecasting.

### 5.4 Recommendations for Agricultural Implementation

Selecting appropriate engineering strategies requires evaluating three criteria: pathogen effector characteristics, crop genetic background, and resource constraints. For pathogens with effector polymorphism evading single NLR recognition (*M. oryzae* AVR‐Pik variants), mismatched NLR pairs provide optimal solutions, achieving recognition of 3 or more effector variants with approximately 65–85% functional compatibility and 15–35% autoactivity [13]. When pathogens deploy helper NLR suppressors (*G. rostochiensis* SPRYSEC15), domain swapping restores immunity by replacing vulnerable domains, yielding ∼60–70% successful receptor function with limited autoactivity in experimental assays [10, 93]. For effectors evolving at recognition interfaces (*P. infestans* AVR3a), targeted mutagenesis expands recognition while preserving specificity, achieving ∼60–75% functional variants and 25–40% instability or autoactivity (Table 2).

Crop genetic background influences strategy selection. Species with large NLR networks (rice, potato, tomato) tolerate all approaches with minimal fitness penalties. Species with smaller repertoires (*Arabidopsis*, legumes) require low‐autoactivity or point‐mutation‐based designs favoring targeted mutagenesis. Polyploid crops (wheat, cotton) accommodate domain swapping due to genetic buffering. Estimated time‐to‐deployment and experimental success rates: targeted mutagenesis (2–3 years, 60–75% success), mismatched pairs (3–5 years, 65–85% success), domain swapping (4‐6 years, 60–70% success), and AI‐guided design (5+ years, experimental stage with unverified durability).

Field validation requires multi‐location trials across at least three locations for minimum three seasons, testing in 2–3 elite breeding lines with side‐by‐side comparisons to commercial varieties. Fitness metrics should include relative yield, days to maturity, biomass, harvest index, disease severity (0–9 scale), and pathogen load via qPCR. Breeding integration uses high‐efficiency genotyping markers (e.g., KASP > 95%), CRISPR editing (eliminates linkage drag), and genomic selection (0.65–0.78 prediction accuracy).

Durability management requires coordinated deployment strategies: 10–20% refuge acreage within ∼500 m of engineered fields; limiting engineered varieties to 40–60% of regional acreage; temporal rotation in 2–3‐year cycles; and annual molecular surveillance of ≥ 50 isolates per region for early resistance‐breakdown detection. Recent empirical advances include Pikobody fusions demonstrating modular design and engineered NLRs (e.g., I2‐I141N) providing broadened resistance in ∼75–85% of field cases [1, 127]. Responsible deployment requires biosecurity frameworks preventing AI tool misuse while ensuring equitable germplasm access for resource‐limited farmers [177]. Successful implementation depends on sustained research, applied breeding programs, science‐based regulatory frameworks, farmer adoption support, public‐private investment, international collaboration on germplasm sharing, and evidence‐based policy supporting innovation and responsible deployment for global food security.

## 6 Conclusion

The integration of structural biology with precision genome editing has transformed plant NLR engineering from empirical breeding to rational molecular design. Three complementary strategies enable targeted enhancement of crop immunity through mismatched allele pairing, domain swapping, and targeted mutagenesis, each addressing distinct vulnerabilities in plant‐pathogen interactions. Mechanistic insights into resistosome assembly and signaling pathways provide design principles validated across diverse crop systems under field conditions.

Despite these advances, several interconnected challenges constrain widespread deployment. Molecular determinants governing fitness‐immunity trade‐offs remain poorly understood, limiting efforts to minimize yield penalties in engineered lines. Pathogen counter‐adaptation through effector variant evolution threatens long‐term durability and demands systematic monitoring. Current screening platforms inadequately predict field performance under realistic stress conditions, creating a deployment bottleneck.

Emerging computational tools offer solutions to these challenges. AlphaFold‐guided engineering and de novo protein design enable capabilities beyond natural variation, while machine learning integration with high‐throughput screening accelerates variant identification and reduces empirical testing burdens. Coordinated field trial networks for systematic durability assessment will bridge the gap between laboratory validation and agricultural implementation. These approaches represent a rational alternative to pesticide‐dependent disease management, providing actionable strategies to combat the 10% to 23% annual yield losses caused by crop pathogens globally. Success requires sustained integration of computational design, experimental validation, and responsible deployment to transform innovations into solutions for global food security.

## Conflicts of Interest

The authors declare no conflicts of interest.
